# Cancer in children of epileptic mothers and the possible relation to maternal anticonvulsant therapy.

**DOI:** 10.1038/bjc.1990.424

**Published:** 1990-12

**Authors:** J. H. Olsen, J. D. Boice, J. F. Fraumeni

**Affiliations:** Danish Cancer Registry, Rosenvaengets, Copenhagen, Denmark.

## Abstract

Cancer incidence among 3,727 offspring of women hospitalised for epilepsy in Denmark between 1933 and 1962 was evaluated in a record-linkage survey with the national cancer registry. The children were identified from hospital charts, population listings, and parish registries. For all children (born before and after their mothers' hospitalisation), no excess of cancer was found in comparison with the general population (49 observed vs 53.8 expected). Among the 2,579 children born after their mothers' first admission for epilepsy, and thus presumably exposed in utero to anticonvulsant drugs, 14 cancers were identified compared to 13.8 expected (relative risk 1.0; 95% confidence interval 0.6-1.7). Contrary to some previous reports, cancers of the brain and nervous system were not significantly increased (3 observed vs 2.2 expected). These data provide no evidence that anticonvulsant drugs are transplacental carcinogens, and indicate that overall increases in risk as high as 80% are unlikely.


*Br. J. Cancer* (1990), **62**, 996–999 © Macmillan Press Ltd., 1990

### Cancer in children of epileptic mothers and the possible relation to maternal anticonvulsant therapy

J.H. Olsen[1], J.D. Boice Jr[2] & J.F. Fraumeni Jr[2]

[1]*Danish Cancer Registry, Rosenvaengets, Hovedvej 35, Box 839, DK-2100, Copenhagen, Denmark; and* [2]*Epidemiology and Biostatistics Program, Division of Cancer Etiology, National Cancer Institute, Bethesda, Maryland, USA.*

**Summary** Cancer incidence among 3,727 offspring of women hospitalised for epilepsy in Denmark between 1933 and 1962 was evaluated in a record-linkage survey with the national cancer registry. The children were identified from hospital charts, population listings, and parish registries. For all children (born before and after their mothers' hospitalisation), no excess of cancer was found in comparison with the general population (49 observed *vs* 53.8 expected). Among the 2,579 children born after their mothers' first admission for epilepsy, and thus presumably exposed *in utero* to anticonvulsant drugs, 14 cancers were identified compared to 13.8 expected (relative risk 1.0; 95% confidence interval 0.6–1.7). Contrary to some previous reports, cancers of the brain and nervous system were not significantly increased (3 observed *vs* 2.2 expected). These data provide no evidence that anticonvulsant drugs are transplacental carcinogens, and indicate that overall increases in risk as high as 80% are unlikely.

The relation of childhood neoplasms to maternal epilepsy and use of anticonvulsants has been suggested in several studies. In a large case–control study of childhood cancer, prenatal exposure to anticonvulsant drugs was implicated (Sanders & Draper, 1979), but subsequent analyses suggested a closer relationship to maternal epilepsy rather than to its treatment (Gilman *et al.*, 1989). A case–control study of childhood brain cancer revealed an association with barbiturate exposure *in utero* and in early life (Gold *et al.*, 1978, 1979), although this finding was not confirmed in two cohort studies (Annegers *et al.*, 1979; Heinonen *et al.*, 1977) or in a recent case–control study (Goldhaber *et al.*, 1990). Case reports have related neuroblastoma to the fetal hydantoin syndrome, a constellation of birth defects among offspring of mothers receiving diphenylhydantoin (phenytoin), but it is not clear if the association is etiologic or a chance event (Koren *et al.*, 1989).

Excess brain tumours have occurred in cohort studies of epileptics treated with anticonvulsant drugs (Clemmesen *et al.*, 1974; White *et al.*, 1979; Shirts *et al.*, 1986; Olsen *et al.*, 1989), but the finding is complicated by the fact that seizures may be an early manifestation of the brain tumour. Further, some studies have suggested relationships of lung cancer following phenobarbital use (Friedman, 1981; Olsen *et al.*, 1989), and lymphomas after phenytoin (Li *et al.*, 1975). In a large follow-up study of cancer among 8,004 epileptic patients exposed to anticonvulsive drugs in Denmark, we found little evidence that phenobarbitone or hydantoins are carcinogenic to humans, although small risks of lung cancer and non-Hodgkin's lymphoma could not be ruled out (Olsen *et al.*, 1989).

To clarify the potential of anticonvulsant drugs to be transplacental carcinogens, we identified the offspring of 3,758 women hospitalised for epilepsy between 1933 and 1962.

### Methods

### *Study population*

The study population included the children of 3,758 female patients admitted for epilepsy to the Filadelfia treatment community in Denmark between 1933 and 1962. Details concerning the identification and selection of the patients have been given in earlier publications (Clemmesen & Hjalgrim-Jensen, 1978; Olsen *et al.*, 1989). Table I shows the age distribution of the female patients at the time of first admission to the epilepsy hospital over three consecutive 10-year calendar periods. Overall, 45% were below age 20 and 38% were ages 20–39 at first admittance, which is also regarded as the time when treatment for epilepsy began.

**Table I** Number of female patients hospitalised for epilepsy between 1933 and 1962 at Filadelfia, Denmark by age and year of admission

| *Age at admission* | *Year of admission* 1933–42 | 1943–52 | 1953–62 | Total | (%) |
|---|---|---|---|---|---|
| 0–19 | 244 | 560 | 906 | 1710 | (45) |
| 20–39 | 408 | 562 | 455 | 1425 | (38) |
| 40–59 | 122 | 189 | 257 | 568 | (15) |
| ⩾60 | 3 | 9 | 43 | 55 | (2) |
| Total | 777 | 1320 | 1661 | 3758 | (100) |

### *Drug exposure*

Up until the 1960s phenobarbital and hydantoins were the principal drugs used to treat epilepsy at Filadelfia. Phenobarbitone was introduced in the early 1920s followed by hydantoins in the 1940s. In the mid-1950s, primidone, which is a barbiturate partly converted to phenobarbitone after ingestion, was introduced for treatment of grand mal seizures. Records from a sample of 130 female epileptic patients were abstracted to obtain more detailed information on drug use. Women hospitalised for epilepsy commonly received daily doses of 100–300 mg of phenobarbitone, or other drugs, and treatment often continued for life (Olsen *et al.*, 1989).

### *Identifying offspring*

A Central Population Register (CPR) was established in Denmark on 1 April 1968, when all citizens were assigned a unique 10-digit personal identification (ID) number. The CPR includes information on vital status, addresses, and ways to identify parents and children, if both were alive on (or after) 1 April 1968 and still living in the same household at that time.

The female epileptics were thus separated into two groups, those with known ID numbers ($n = 3{,}066$; 82%), and those who died before 1 April 1968 without an ID number ($n = 692$; 18%). By means of computerised record linkage with the CPR, a search was made for all children born between 1 April 1968 and 31 December 1986.

Offspring born before the inception of the CPR were identified through the 276 local population registers in Denmark. Because deceased children are not 'transferred' to a

Correspondence: J.H. Olsen.
Received 20 April 1990; and in revised form 17 July 1990.

new population register when a family changes its place of residence, complete residential histories were searched for all female patients over age 17 on 1 April 1968. This tracing was relatively complete in that residential coverage was determined for 82% of the time (on average) a woman was alive between the ages of 18 and 50 years. Local population registries were then contacted for information on children. No attempt was made to trace offspring of female epileptics deceased before 1 April 1968.

An average of 1.2 children per female epileptic was identified, 3,727 children overall (Table II). The number of nulliparous women was high in comparison with the general population, 47% versus approximately 20% (Ewertz & Jensen, 1984). Among those with children, the family size was fairly similar to the national average (Ewertz & Duffy, 1988).

*Cancer incidence and analysis*

A complete description and evaluation of the Danish Cancer Registry have been given earlier (Jensen *et al.*, 1985). Records on the offspring of female epileptics were linked with the files of the Cancer Registry following a previously established procedure (Jensen, 1980). The period of observation for calculation of the risk of developing a cancer began at date of birth or 1 January 1943 (when the registry began), whichever occurred later. The end of the period was taken as the date of last contact, i.e. the date of death, emigration, or 31 December 1986, for those known to be alive at the study closing date. The number of expected malignancies was calculated by applying the cancer incidence rates by site, sex, 5-year age and 5-year time periods to the appropriate person-years under observation (Monson, 1974). Statistical methods used were based on the assumption that the observed numbers of cancer cases in any specific category will follow a Poisson distribution. Tests of significance and confidence intervals (CI) for the relative risk (RR), taken as the ratio of observed to expected individual cancers, were calculated with the use of the exact Poisson probabilities when the observed number of cases was small; otherwise, an accurate asymptotic approximation was used (Rothman & Boice, 1979).

### Results

The survey on drug use among a sample ($n$ = 130) of female epileptic patients indicated that 76% had been treated with phenobarbitone, 59% with hydantoins, and 30% with primidone. Table III gives the proportion of female epileptics who ever used one or more of the specified groups of drug during four consecutive calendar periods, and the typical daily doses prescribed. For 13% of the women in the sample, evidence of anticonvulsant therapy was not apparent from their hospital records.

Among the 3,727 offspring included in the study (1,933 boys and 1,794 girls), 3,395 (91%) were known to be alive at the end of the study period (31 December 1986), 243 (7%) had died, and 89 (2%) had emigrated. In the total group of children, 1,148 were born before the initial admittance of the mother to Filadelfia, and 2,579 after hospitalisation. A total

**Table II** Some characteristics of the 3,727 identified offspring of 3,066 epileptic women[a]

| *Characteristics* | *Number* | *Per cent* |
|---|---|---|
| Verified children | | |
| Boys | 1933 | 51.9 |
| Girls | 1794 | 48.1 |
| Total | 3727 | 100.0 |
| Year of birth | | |
| 1912–36 | 387 | 10.4 |
| 1937–61 | 1840 | 49.4 |
| 1962–86 | 1500 | 40.2 |
| Vital status (31 December 1986) | | |
| Alive | 3395 | 91.1 |
| Deceased | 243 | 6.5 |
| Emigrated | 89 | 2.4 |
| Number of children per epileptic woman | | |
| None | 1438 | 46.9 |
| 1 | 447 | 14.6 |
| 2 | 637 | 20.8 |
| 3 | 331 | 10.8 |
| 4 or more | 213 | 6.9 |

[a]The mothers of these children had to be alive on 1 April 1968 for their offspring to be included in the study.

of 42,154 person-years of follow-up were accumulated in the former group and 57,741 in the latter, for an average follow-up of 37.5 years (maximum, 65) and 22.4 years (maximum, 50), respectively.

Overall, 49 cancers were identified compared to 53.8 expected (RR 0.91; 95% CI 0.7–1.2). Among the group of offspring born before the mothers' initial admission to Filadelfia, 35 developed a cancer compared to 40.0 expected (RR 0.88; 95% CI 0.6–1.2). No significant increase in risk for any cancer was found, although small excesses were noted for lung cancer (7 versus 3.2) and nonmelanoma skin cancer (7 versus 3.8). As shown in Table IV, 14 cancers occurred among offspring born after the mothers' initial admission compared to 13.8 expected (RR 1.0; 95% CI 0.6–1.7). No excess risk was observed for any specific cancer. Table V shows the tumour types, sex, age at diagnosis, and maternal drug histories of children (born after their mothers' initial admission) who developed cancer. Two of the three brain cancer cases were astrocytomas; however, no anticonvulsants were given during pregnancy for one case, and in the other no information on therapy was available. One of the two children with leukaemia also had Down's syndrome.

### Discussion

Since 1971 when prenatal exposure to synthetic estrogens was linked to clear cell adenocarcinomas of the vagina and cervix (Herbst *et al.*, 1971), there has been great interest in whether other drugs pose transplacental hazards. Some suspicion has centered on phenytoin, because of clinical reports of neuroblastoma with the fetal hydantoin syndrome. The relation of prenatal barbiturate exposure to childhood brain tumours was raised by one study (Gold *et al.*, 1978, 1979), although not confirmed by others (Annegers *et al.*, 1979; Heinonen *et*

**Table III** Proportion of female patients ever taking anticonvulsant drugs during four specific time periods obtained from a sample of 130 patients[a]

| *Anticonvulsants ever taken* | *Time period of treatment* 1933–9 % | 1940–9 % | 1950–9 % | 1960–9 % | *Typical daily doses (mg)* |
|---|---|---|---|---|---|
| Phenobarbitone | 94 | 80 | 76 | 59 | 200 |
| Hydantoins | 3 | 42 | 55 | 50 | 200 |
| Primidone | 0 | 0 | 28 | 27 | 500 |
| Carbamazepine | 0 | 0 | 0 | 5 | 800 |
| Other drugs | 3 | 9 | 9 | 15 | – |

[a]May sum up to more than 100% since some patients received more than one drug within each time period.

**Table IV** Observed (O) and expected (E) incident cancers between 1943 and 1986 among 2,579 children born after admission of their mothers to the Filadelfia hospital

| *Site* | *O* | *E* | *O/E* | *(95% CI)* |
|---|---|---|---|---|
| Cervix uteri | 1 | 0.79 | 1.3 | (0.0–7.0) |
| Breast | 0 | 0.91 | 0.0 | (0.0–4.1) |
| Testis | 2 | 1.34 | 1.5 | (0.2–5.4) |
| Melanoma of skin | 2 | 0.79 | 2.6 | (0.3–9.2) |
| Other skin | 1 | 0.74 | 1.4 | (0.1–7.0) |
| Brain and nervous system | 3 | 2.17 | 1.4 | (0.3–4.0) |
| Bone | 1 | 0.38 | 2.7 | (0.0–15) |
| Hodgkin's disease | 1 | 0.77 | 1.3 | (0.0–7.2) |
| Non-Hodgkin's lymphoma | 0 | 0.64 | 0.0 | (0.0–5.8) |
| Leukaemia | 2 | 2.08 | 1.0 | (0.1–3.5) |
| Other | 1 | 3.16 | 0.3 | (0.0–1.8) |
| All cancers | 14 | 13.77 | 1.0 | (0.6–1.7) |

*al.*, 1977; Goldhaber *et al.*, 1990). Further interest has been aroused by reports that after postnatal exposure hydantoins may be related to lymphoma (Li *et al.*, 1975), and phenobarbitone to lung cancer (Friedman, 1981).

In this cohort study of 3,727 children born to epileptic mothers, we found no evidence of increased cancer risk. A maternal history of hospitalisation for epilepsy was used as a proxy for fetal drug exposure, and cancer was evaluated in offspring born both before and after hospitalisation. No forms of cancer could be linked to either a maternal history of epilepsy or exposure *in utero* to anticonvulsant drugs. However, despite the relatively large number of exposed offspring in our study, the number of observed cancers was relatively small, and the power to detect the relatively small relative risks suggested in several previous studies was low. While two-fold risks for all cancer sites combined can be reasonably excluded in our series, lower level risks cannot. For specific cancer sites, the upper confidence limits indicate that three-fold and greater risks would not be incompatible with the current observations. In addition, we could not obtain complete drug histories on all individuals, only details from a sample and from those mothers whose children developed malignancy. Also, about 13% of the women who were hospitalised for epilepsy apparently received little or no anticonvulsant treatment, so that fetal exposures are less certain. On the other hand, we had relatively complete identification of children born to epileptic mothers and relatively complete ascertainment of cancer incidence through linkage with available national record systems. The period of observation was long, and cancers through early adulthood (ages 24 through 53 years) could be identified for the majority of offspring.

Prenatal exposures to anticonvulsants were likely to be heaviest among the 2,579 children born after hospitalisation of their mothers for epilepsy. This subgroup showed no excess risk of any form of cancer. Although the developing fetus may be especially sensitive to the effects of carcinogenic exposures, these data suggest that if anticonvulsants are carcinogenic, our study size was too small to detect small increases in risk, the cumulative dose at critical periods was too low, or the observational period was too short to accommodate the latency for certain forms of cancer.

**Table V** Cancers occurring among children born after the admission of their mothers to the Filadelfia hospital

| *Case no.* | *Site and type* | *Sex* | *Age at diagnosis* | *Anticonvulsants used during pregnancy (daily dosage)* |
|---|---|---|---|---|
| 1 | Medulloblastoma of cerebellum | M | 9 | Phenobarbitone (100 mg)<br>Phenytoin (300 mg) |
| 2 | Pilocystic astrocytoma of brain | F | 6 | No indication of use |
| 3 | Astrocytoma of brain | M | 12 | Information not available |
| 4 | Testis, seminoma | M | 29 | Phenobarbitone (200 mg)<br>Phenytoin (300 mg) |
| 5 | Testis, teratocarcinoma | M | 26 | Phenytoin (200 mg)<br>Primidone (250 mg) |
| 6 | Embryonal carcinoma of retroperitoneal tissue | M | 20 | Phenytoin (300 mg)<br>Primidone (750 mg) |
| 7 | Acute lymphatic leukaemia[a] | F | 7 | Phenobarbitone (250 mg)<br>Phenytoin (50 mg) |
| 8 | Acute myeloid leukaemia | F | 3 | Phenytoin (200 mg)<br>Ethosuximide (750 mg) |
| 9 | Malignant lymphogranulomatosis | M | 2 | No indication of use |
| 10 | Skin melanoma, NOS | F | 25 | Phenytoin (100 mg) |
| 11 | Skin melanoma, superficial spreading type | F | 26 | No indication of use |
| 12 | Basal cell carcinoma of skin | F | 37 | No indication of use |
| 13 | Osteogenic sarcoma of femur | M | 12 | No indication of use |
| 14 | Cervix uteri, squamous cell carcinoma | F | 21 | Phenobarbitone (150 mg) |

[a]Child also had Down's syndrome.

### References

ANNEGERS, J.F., KURLAND, L.T. & HAUSER, W.A. (1979). Brain tumors in children exposed to barbiturates. *J. Natl Cancer Inst.*, **63**, 3.

CLEMMESEN, J., FULGSANG-FREDERIKSEN, V. & PLUM, C.M. (1974). Are anticonvulsants oncogenic? *Lancet*, **i**, 705.

CLEMMESEN, J. & HJALGRIM-JENSEN, S. (1978). Is phenobarbital carcinogenic? A follow-up of 8078 epileptics. *Ecotoxicol. Environ. Safety*, **1**, 457.

EWERTZ, M. & JENSEN, O.M. (1984). Trends in the incidence of cancer of the corpus uteri in Denmark, 1943–1980. *Am. J. Epidemiol.*, **119**, 725.

EWERTZ, M. & DUFFY, S.W. (1988). Risk of breast cancer in relation to reproductive factors in Denmark. *Br. J. Cancer*, **58**, 99.

FRIEDMAN, G.D. (1981). Barbiturates and lung cancer in humans. *J. Natl Cancer Inst.*, **67**, 291.

GILMAN, E.A., WILSON, L.M., KNEALE, G.W. & WATERHOUSE, J.A. (1989). Childhood cancers and their association with pregnancy drugs and illnesses. *Paediatr. Perinat. Epidemiol.*, **3**, 66.

GOLD, E., GORDIS, L., TONASCIA, J. & SZKLO, M. (1978). Increased risk of brain tumors in children exposed to barbiturates. *J. Natl Cancer Inst.*, **61**, 1031.

GOLD, E., GORDIS, L., TONASCIA, J. & SZKLO, M. (1979). Brain tumors in children exposed to barbiturates (letter). *J. Natl Cancer Inst.*, **63**, 3.

GOLDHABER, M.K., SELBY, J.V. & QUESENBERRY, C.P. (1990). Exposure to barbiturates *in utero* and during childhood and risk of intracranial and spinal cord tumours. *Cancer Res.*, **50**, 4600.

HEINONEN, O.P., SLONE, D. & SHAPIRO, S. (1977). *Birth Defects and Drugs in Pregnancy*. Publishing Sciences Group: Littleton, MA.

HERBST, A.L., ULFELDER, H. & POSKANZER, D.C. (1971). Adenocarcinoma of the vagina. Association of maternal stilbestrol therapy with tumor appearance in young women. *N. Engl. J. Med.*, **284**, 878.

JENSEN, O.M. (1980). *Cancer Morbidity and Causes of Death among Danish Brewery Workers*. IARC: Lyon.

JENSEN, O.M., STORM, H.H. & JENSEN, H.S. (1985). Cancer registration in Denmark and the study of multiple primary cancer, 1943-1980. *Natl Cancer Inst. Monogr.*, **68**, 245.

KOREN, G., DEMITRAKONDIS, D., WEKSBERG, R. & ? others (1989). Neuroblastoma after prenatal exposure to phenytoin: cause and effect? *Teratology*, **40**, 157.

LI, F.P., WILLARD, D.R., GOODMAN, R. & ? others (1975). Malignant lymphoma after diphenylhydantoin (dilantin) therapy. *Cancer*, **36**, 1359.

MONSON, R.R (1974). Analysis of relative survival and proportional mortality. *Comput. Biomed. Res.*, **7**, 325.

OLSEN, J.H., BOICE, J.D., JENSEN, J.P.A. & FRAUMENI, J.F. (1989). Cancer among epileptic patients exposed to anticonvulsant drugs. *J. Natl Cancer Inst.*, **81**, 803.

ROTHMAN, K.J. & BOICE, J.D. Jr (1979). *Epidemiologic Analysis with a Programmable Calculator*. DHHS (NIH) Publication No. 79-1649, US Government Printing Office: Washington, DC.

SANDERS, B.M. & DRAPER, G.J. (1979). Childhood cancer and drugs in pregnancy. *Br. Med. J.*, **i**, 717.

SHIRTS, S.B., ANNEGERS, J.F., HAUSER, W.A. & ? others (1986). Cancer incidence in a cohort of patients with seizure disorders. *J. Natl Cancer Inst.*, **77**, 83.

WHITE, S.J., MCLEAN, A.E.M. & HOWLAND, C. (1979). Anticonvulsant drugs and cancer: a cohort study in patients with severe epilepsy. *Lancet*, **ii**, 458.


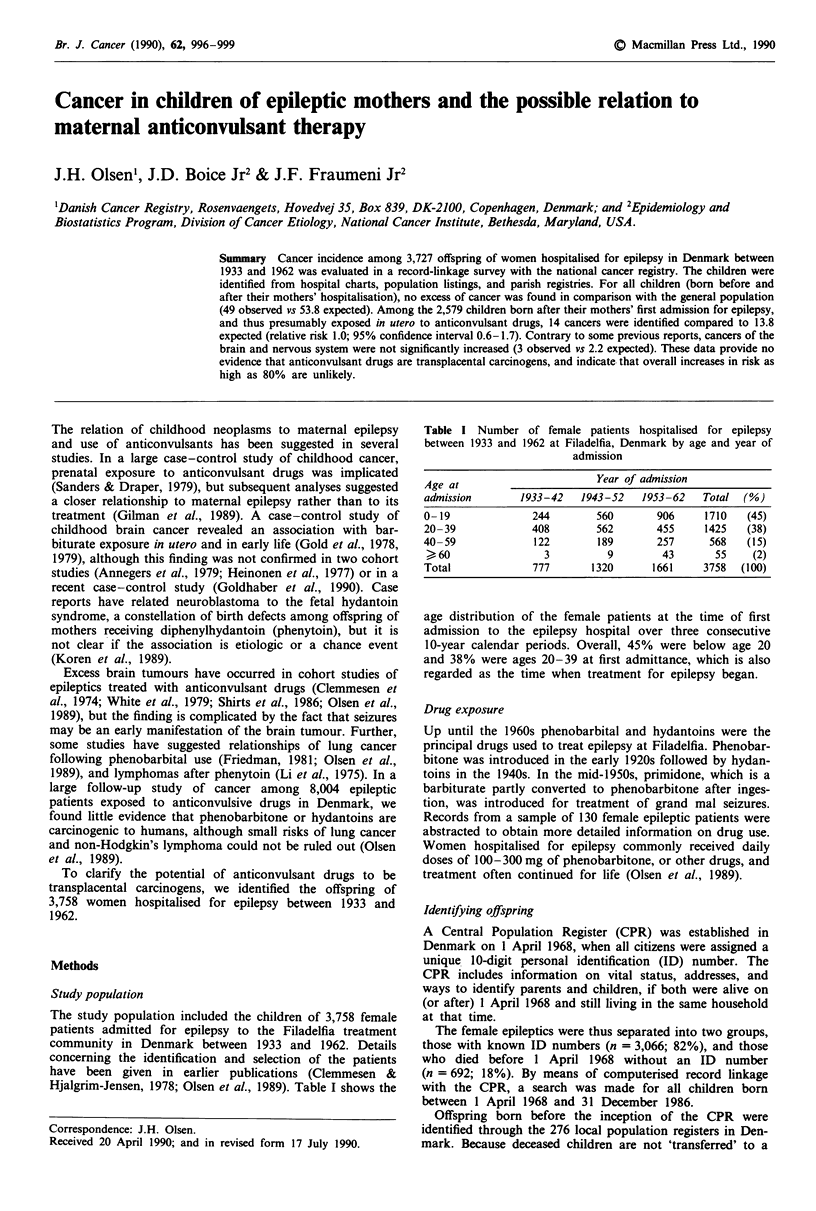

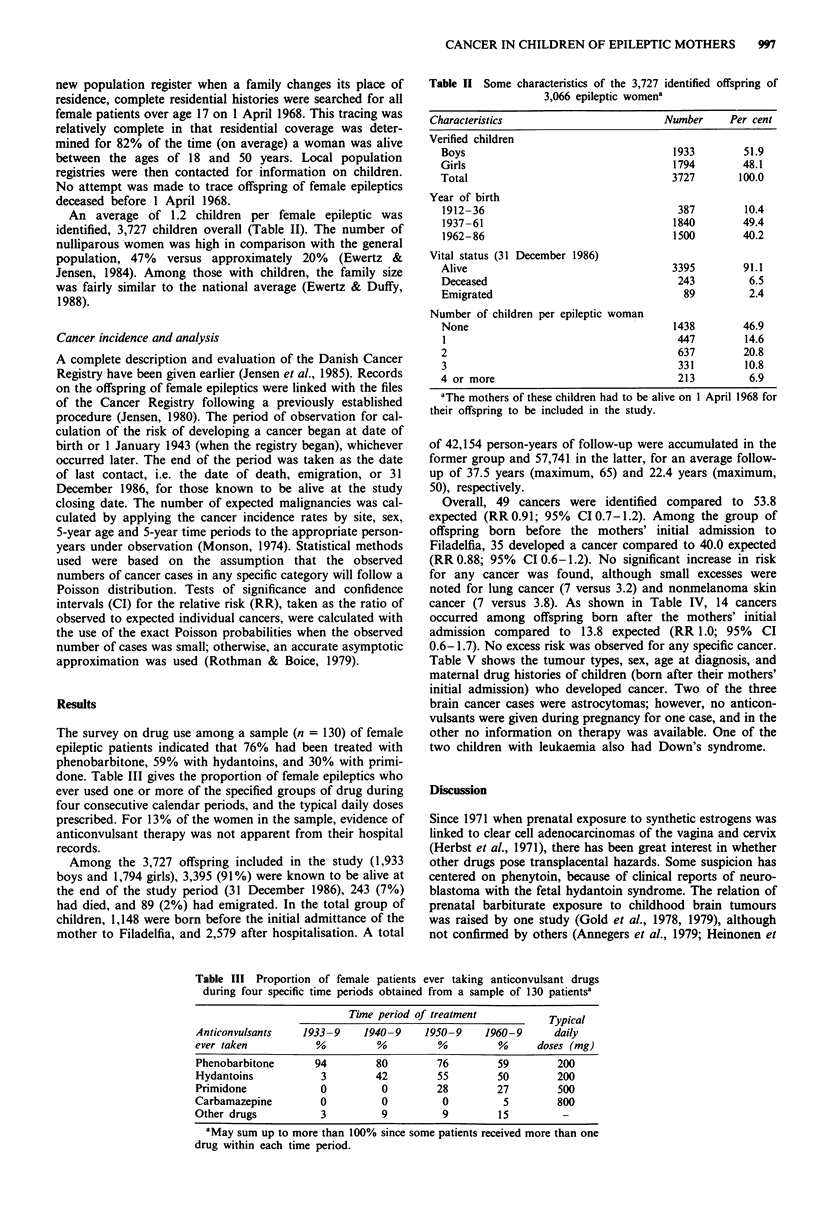

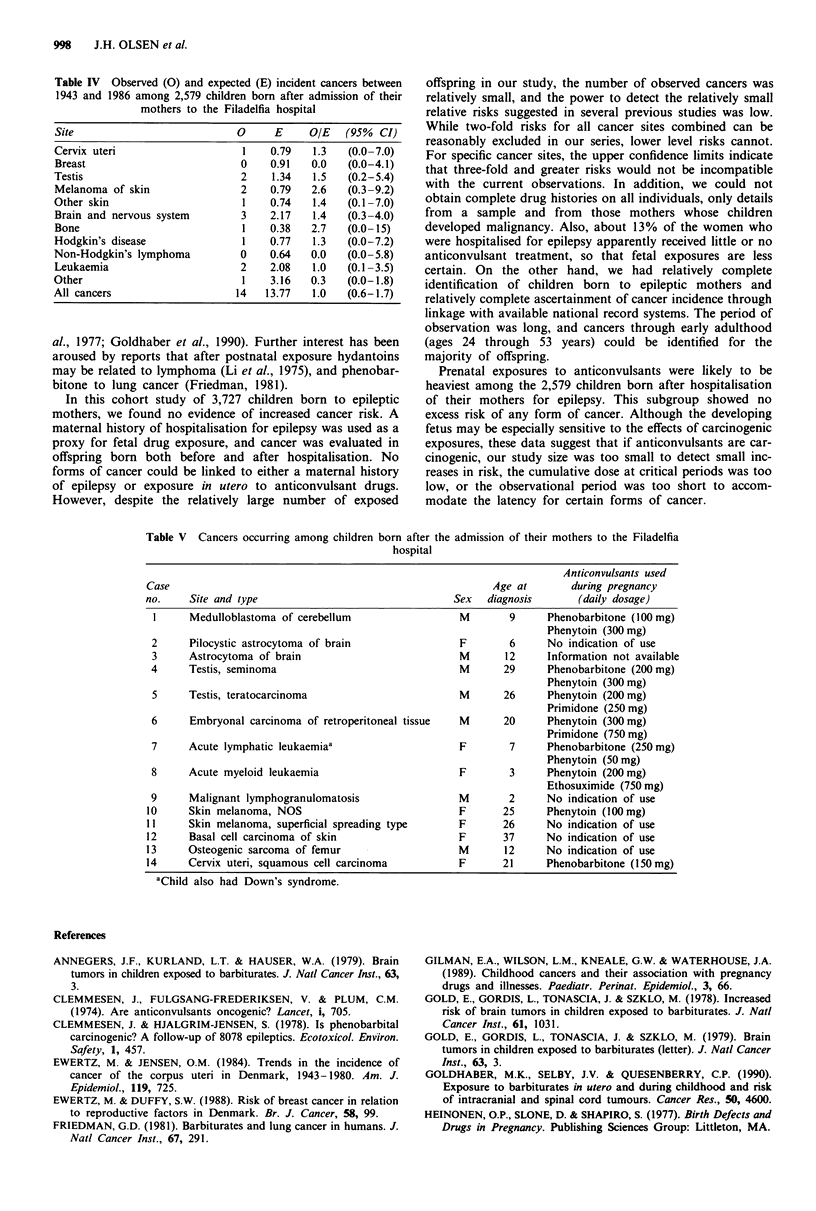

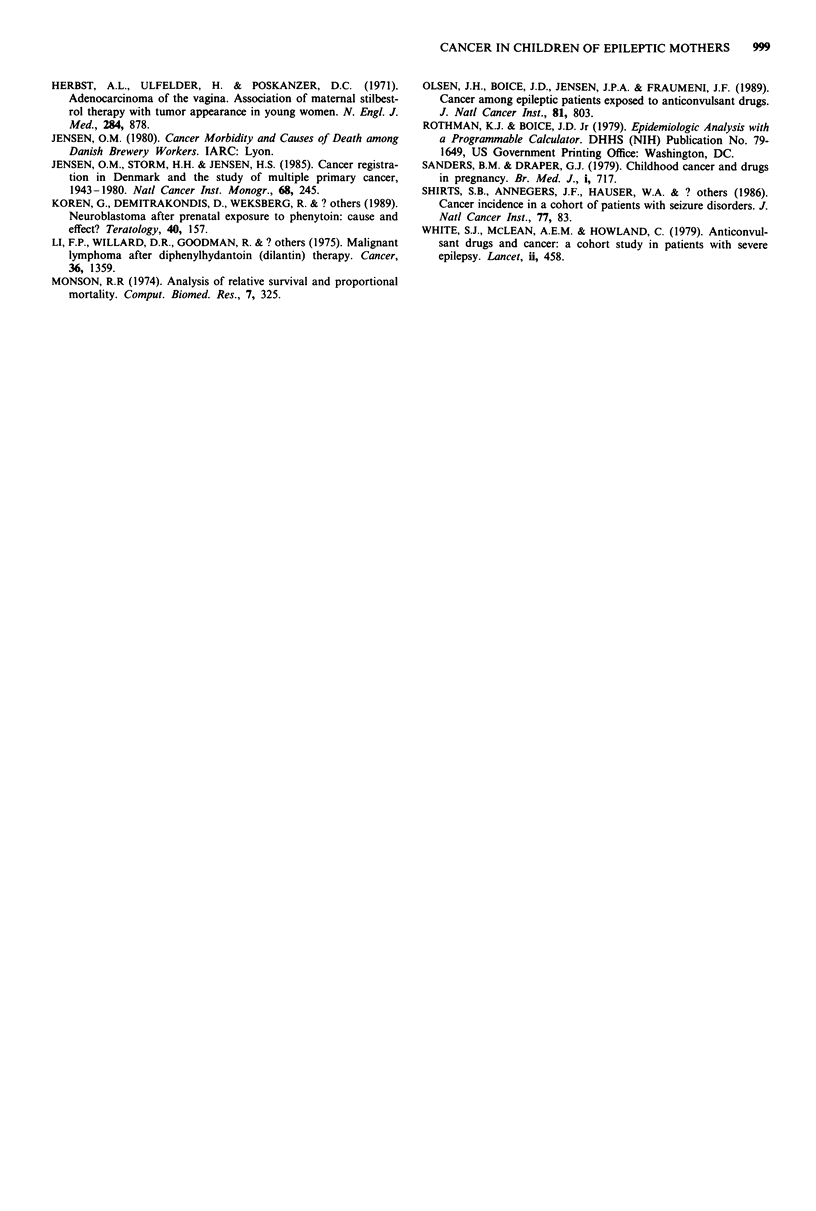


## References

[OCR_00395] Annegers J. F., Kurland L. T., Hauser W. A. (1979). Brain tumors in children exposed to barbiturates.. J Natl Cancer Inst.

[OCR_00400] Clemmesen J., Fuglsang-Frederiksen V., Plum C. M. (1974). Are anticonvulsants oncogenic?. Lancet.

[OCR_00404] Clemmesen J., Hjalgrim-Jensen S. (1978). Is phenobarbital carcinogenic? A follow-up of 8078 epileptics.. Ecotoxicol Environ Saf.

[OCR_00414] Ewertz M., Duffy S. W. (1988). Risk of breast cancer in relation to reproductive factors in Denmark.. Br J Cancer.

[OCR_00409] Ewertz M., Jensen O. M. (1984). Trends in the incidence of cancer of the corpus uteri in Denmark, 1943-1980.. Am J Epidemiol.

[OCR_00418] Friedman G. D. (1981). Barbiturates and lung cancer in humans.. J Natl Cancer Inst.

[OCR_00422] Gilman E. A., Wilson L. M., Kneale G. W., Waterhouse J. A. (1989). Childhood cancers and their association with pregnancy drugs and illnesses.. Paediatr Perinat Epidemiol.

[OCR_00432] Gold E. B., Gordis L., Tonascia J. A., Szklo M. (1979). Brain tumors in children exposed to barbiturates.. J Natl Cancer Inst.

[OCR_00427] Gold E., Gordis L., Tonascia J., Szklo M. (1978). Increased risk of brain tumors in children exposed to barbiturates.. J Natl Cancer Inst.

[OCR_00437] Goldhaber M. K., Selby J. V., Hiatt R. A., Quesenberry C. P. (1990). Exposure to barbiturates in utero and during childhood risk of intracranial and spinal cord tumors.. Cancer Res.

[OCR_00447] Herbst A. L., Ulfelder H., Poskanzer D. C. (1971). Adenocarcinoma of the vagina. Association of maternal stilbestrol therapy with tumor appearance in young women.. N Engl J Med.

[OCR_00457] Jensen O. M., Storm H. H., Jensen H. S. (1985). Cancer registration in Denmark and the study of multiple primary cancers, 1943-80.. Natl Cancer Inst Monogr.

[OCR_00462] Koren G., Demitrakoudis D., Weksberg R., Rieder M., Shear N. H., Sonely M., Shandling B., Spielberg S. P. (1989). Neuroblastoma after prenatal exposure to phenytoin: cause and effect?. Teratology.

[OCR_00467] Li F. P., Willard D. R., Goodman R., Vawter G. (1975). Malignant lymphoma after diphenylhydantoin (dilantin) therapy.. Cancer.

[OCR_00472] Monson R. R. (1974). Analysis of relative survival and proportional mortality.. Comput Biomed Res.

[OCR_00476] Olsen J. H., Boice J. D., Jensen J. P., Fraumeni J. F. (1989). Cancer among epileptic patients exposed to anticonvulsant drugs.. J Natl Cancer Inst.

[OCR_00486] Sanders B. M., Draper G. J. (1979). Childhood cancer and drugs in pregnancy.. Br Med J.

[OCR_00495] White S. J., McLean A. E., Howland C. (1979). Anticonvulsant drugs and cancer. A cohort study in patients with severe epilepsy.. Lancet.

